# Risk Factors for *Bartonella* species Infection in Blood Donors from Southeast Brazil

**DOI:** 10.1371/journal.pntd.0004509

**Published:** 2016-03-21

**Authors:** Pedro Paulo Vissotto de Paiva Diniz, Paulo Eduardo Neves Ferreira Velho, Luiza Helena Urso Pitassi, Marina Rovani Drummond, Bruno Grosselli Lania, Maria Lourdes Barjas-Castro, Stanley Sowy, Edward B. Breitschwerdt, Diana Gerardi Scorpio

**Affiliations:** 1 College of Veterinary Medicine, Western University of Health Sciences, Pomona, California, United States of America; 2 Division of Dermatology, Department of Medicine, State University of Campinas, Medical School (UNICAMP), Campinas, São Paulo, Brazil; 3 Hematologia e Hemoterapia (HEMOCENTRO), Department of Medicine, State University of Campinas (UNICAMP), Campinas, São Paulo, Brazil; 4 Intracellular Pathogens Research Laboratory, College of Veterinary Medicine, North Carolina State University, Raleigh, North Carolina, United States of America; 5 Department of Molecular and Comparative Pathobiology, Johns Hopkins University School of Medicine, Baltimore, Maryland, United States of America; 6 Department of Biomedical Sciences, Ross University School of Veterinary Medicine, Basseterre, St. Kitts and Nevis, West Indies; Texas A&M University, UNITED STATES

## Abstract

Bacteria from the genus *Bartonella* are emerging blood-borne bacteria, capable of causing long-lasting infection in marine and terrestrial mammals, including humans. *Bartonella* are generally well adapted to their main host, causing persistent infection without clinical manifestation. However, these organisms may cause severe disease in natural or accidental hosts. In humans, *Bartonella* species have been detected from sick patients presented with diverse disease manifestations, including cat scratch disease, trench fever, bacillary angiomatosis, endocarditis, polyarthritis, or granulomatous inflammatory disease. However, with the advances in diagnostic methods, subclinical bloodstream infection in humans has been reported, with the potential for transmission through blood transfusion been recently investigated by our group. The objective of this study was to determine the risk factors associated with *Bartonella* species infection in asymptomatic blood donors presented at a major blood bank in Southeastern Brazil. Five hundred blood donors were randomly enrolled and tested for *Bartonella* species infection by specialized blood cultured coupled with high-sensitive PCR assays. Epidemiological questionnaires were designed to cover major potential risk factors, such as age, gender, ethnicity, contact with companion animals, livestock, or wild animals, bites from insects or animal, economical status, among other factors. Based on multivariate logistic regression, bloodstream infection with *B*. *henselae* or *B*. *clarridgeiae* was associated with cat contact (adjusted OR: 3.4, 95% CI: 1.1–9.6) or history of tick bite (adjusted OR: 3.7, 95% CI: 1.3–13.4). These risk factors should be considered during donor screening, as bacteremia by these *Bartonella* species may not be detected by traditional laboratory screening methods, and it may be transmitted by blood transfusion.

## Author Summary

Bacteria from the genus *Bartonella* are capable of causing long-lasting infection. Despite the fact that these bacteria may cause several diseases such as cat scratch disease, trench fever, and infection of cardiac valves, which can be fatal, they may also cause asymptomatic infection in humans. Several blood-sucking arthropods have been suggested or confirmed as responsible for transmitting these bacteria, including sandflies, body lice, fleas, ticks, and keds. In this study, 500 asymptomatic human blood donors from Brazil were screened for infection with species of *Bartonella* by blood culture coupled with molecular detection and genetic sequencing, and risk factors associated with such infection were identified. In this population, contact with cats and history of tick bite were significantly associated with human infection by *Bartonella* species. Since laboratory screening of donated blood for the presence of *Bartonella* species is not generally performed by blood banks, these risk factors should be should be considered during donor screening in order to avoid transmission of *Bartonella* species by blood transfusion.

## Introduction

*Bartonella* species are fastidious alpha-proteobacteria with worldwide distribution. To date, at least 15 species have been associated with human infections, with at least eight species also capable of infecting dogs and cats. Several blood-sucking arthropods have been suggested or confirmed as vectors for this genus, including sandflies, body lice, fleas, ticks, and keds [1]. In humans, select species of *Bartonella* were confirmed as etiologic agents of cat scratch disease (CSD), trench fever, bacillary angiomatosis and Oroya fever [2]. Moreover, *Bartonella* infections have also been documented by culture or molecular methods in human cases of endocarditis, myocarditis, polyarthritis and granulomatous inflammatory disease [1, 2]. In countries throughout the world, most diseases associated with *Bartonella* species infection are not reportable; therefore, incidence data is scarce. One study performed at the end of the 20^th^ century estimated that 22,000 new cases of cat scratch disease appear every year in the United States, and roughly 10% of these infections were thought to require hospitalization [3].

*Bartonella* species cause chronic and intermittent intra-erythrocytic bacteremia and infect endothelial cells of both incidental and natural reservoir hosts. The establishment of chronic, stealth infection is achieved by evasion of innate immune responses. These include resistance to complement activation, antigenic variation of surface proteins, and inhibition of host cell apoptosis [4]. Consequently, subclinical bloodstream infection in humans has been reported [5], supporting the fact that these bacteria will not follow Koch’s postulates for disease causation [6].

Asymptomatic *Bartonella* species infection poses a hazard for blood recipients because fast, sensitive and specific diagnostic tests are not currently available for donor screening. To the authors’ knowledge, no prophylactic measures are currently in place to prevent collection and transfusion of blood and blood products contaminated with species of *Bartonella* worldwide. Recently, using a combination of culture methods and PCR assays, we documented *Bartonella henselae* and *Bartonella clarridgeiae* infection of blood samples from 16 asymptomatic blood donors at a large blood bank center in Southeast Brazil [5]. Risk factors for *Bartonella* species exposure in blood donors were initially evaluated in one study from Sweden using serology methods [7]. However, antibody detection has limited predictive value in confirming or excluding *Bartonella* species bacteremia in humans and animals [6, 8–10]. Furthermore, higher vector activity is expected in tropical and sub-tropical regions of the world. Therefore, this study objective was to determine which risk factors are associated with *Bartonella* species infection in asymptomatic blood donors from a population in Campinas, Sao Paulo State, Brazil.

## Methods

### Ethics statement

The Institutional Review Board of the State University of Campinas, Brazil approved this study under protocol number CEP 122/2005. The voluntary blood donors presented at the Blood Bank (HEMOCENTRO) of the State University of Campinas (UNICAMP), Brazil, and were enrolled after inform written consent was obtained.

### Sample collection and diagnostic techniques

We collected blood from five hundred apparently healthy voluntary blood donors in this cross sectional study. Sample size was determined with an alpha of 0.05, power of 0.8, and estimated prevalence of 5%, as previously reported in a serology-based study [11]. Donors were selected through convenience sampling, with inclusion and exclusion criteria following the current international standards for blood bank donor selection [12].

*Bartonella* species infection from the bloodstream was detected based on enrichment blood culture in a liquid growth medium (*Bartonella* alpha-Proteobacteria growth medium-BAPGM), coupled with isolation in solid medium and *Bartonella*-specific DNA amplification by PCR, followed by DNA sequencing to confirm species identification [8].

### Epidemiological data collection

A standardized epidemiological questionnaire was delivered to each blood donor participant. The interviewer had no knowledge of the diagnostic test results, as *Bartonella* species testing was performed subsequent to interviews. The following information was captured: gender; self-reported ethnicity as African-American, native Indian, Caucasian, Asian or multi-racial; average monthly income represented by multiples of Brazilian monthly minimal wage; occupational animal exposure (veterinary professionals and others with direct animal exposure such as veterinary assistants, ranchers, biologists, and volunteers at shelters); contact with cats, dogs, other companion animals, livestock or wildlife (including handling animals, bedding, waste or sharing the same environment); bites from dogs, cats, and other animals; arthropod bites caused by ticks, fleas, or other insects; previous blood transfusion; previous history of blood donation; and presence of tattoos. Past history of contact with animals, animal bites, and/or arthropod bites (defined as more than one year after contact) were also recorded.

### Data analysis

Potential risk factors were first compared in a univariate analysis using Fisher’s exact test or the Fisher-Freeman-Halton test. All risk factors significant at the *p*< 0.25 level were entered into a stepwise logistic regression model, and variables significant to *p*< 0.05 were retained. Univariate odds ratios (OR), adjusted odds ratios (aOR), and 95% confidence intervals (CI) were calculated. Variables with collinearity were removed from the multivariate analysis. Statistical analyses were performed using JMP Pro 10 for Windows (SAS Institute Inc., Cary, NC).

## Results

*Bartonella* species bloodstream infection was detected in 16/500 blood donors (3.2%). DNA amplification and sequencing identified *B*. *henselae* in 15 blood donors (3%) and *B*. *clarridgeiae* in one donor (0.2%), which was previously reported [5, 13]. *B*. *henselae* bacteremia was also confirmed in six donors by bacterial isolation. The univariate and multivariate analyses of risk factors between blood donors infected with *Bartonella* species and uninfected subjects are provided in Tables 1 and 2. With univariate analysis, a professional with animal exposure was seven times more likely to be infected with *Bartonella* species than blood donors working in all other professions. When significant variables were entered into the multivariate logistic regression model, collinearity between animal-related professions and cat contact was documented; therefore, profession was not maintained in the final model. Adjusted odds ratio indicated that subjects with cat contact, or past history of tick bite, were approximately 3 to 4 times more likely to have a *Bartonella* species infection than donors without cat contact or lack of history of tick bite (Table 2).

**Table 1:** Univariate analysis of risk factors between blood donors with *Bartonella* infection detected by enrichment PCR, compared with uninfected subjects^a^.

Characteristic	Categories	*Bartonella* infected (N = 16) N (%)	Non-infected (N = 484) N (%)	Univariate *p* value^b^
Gender	Female	5 (31.3)	177 (36.7)	0.795
	Male	11 (68.7)	306 (63.3)	
Ethnicity	African American	2 (13.3)	55 (11.6)	0.988
	Amerindian	0 (0)	4 (0.8)	
	Caucasian	11 (73.3)	349 (73.6)	
	East Asian	0 (0)	5 (1.1)	
	Multiracial	2 (13.3)	61 (12.8)	
Average montly income (in multiples of the Brazilian montly minimal wage^c^)	Less than 1	0 (0)	2 (0.4)	0.611
	1–2	1 (6.3)	84 (17.4)	
	2–5	10 (62.5)	233 (48.4)	
	5–10	3 (18.7)	104 (21.6)	
	10–15	2 (12.5)	35 (7.28)	
	Above 15	0 (0)	23 (4.7)	
Occupational animal exposure	Yes	2 (13.3)	10 (2.1)	0.048*
	No	13 (86.7)	469 (97.9)	
Contact with any animal	Yes	14 (87.5)	366 (75.6)	0.379
	No	2 (12.5)	118 (24.4)	
Contact with dogs	Yes	13 (81.3)	325 (67.3)	0.289
	No	3 (18.7)	158 (32.7)	
Contact with cats	Yes	6 (37.5)	66 (13.7)	0.018*
	No	10 (62.5)	417 (86.3)	
Contact with livestock	Yes	4 (25.0)	69 (14.3)	0.270
	No	12 (75.0)	415 (85.7)	
Contact with wildlife	Yes	1 (6.3)	12 (2.5)	0.348
	No	15 (93.7)	472 (97.5)	
Past contact with animals	Yes	9 (52.3)	324 (66.9)	0.422
(>1 year after contact)	No	7 (43.7)	160 (33.1)	
All animal bites	Yes	4 (25.0)	61 (12.6)	0.142
	No	12 (75.0)	423 (87.4)	
Dog bites	Yes	4 (25.0)	46 (9.5)	0.065
	No	12 (75.0)	438 (90.5)	
Cat bites	Yes	1 (6.3)	17 (3.5)	0.449
	No	15 (93.7)	467 (96.5)	
Other animal bite	Yes	0 (0)	2 (0.4)	1.000
	No	16 (100)	482 (99.6)	
Past animal bite	Yes	9 (56.3)	218 (45.0)	0.448
(>1 year after bite)	No	7 (43.7)	266 (55.0)	
Arthropod bite	Yes	4 (25.0)	187 (38.6)	0.309
(<1 year after bite)	No	12 (75.0)	297 (61.4)	
Flea bite	Yes	0 (0)	4 (0.8)	1.000
	No	15 (100)	479 (99.2)	
Tick bite	Yes	0 (0)	29 (6.0)	0.615
	No	16 (100)	453 (94.0)	
Other insect bite	Yes	1 (7.1)	114 (26.4)	0.129
	No	13 (92.9)	318 (73.6)	
Past arthropod bite	Yes	13 (81.3)	286 (59.2)	0.117
(>1 year after bite)	No	3 (18.7)	197 (40.8)	
Past flea bite	Yes	3 (18.8)	45 (9.3)	0.192
	No	13 (81.2)	438 (90.7)	
Past tick bite	Yes	12 (75.0)	207 (42.9)	0.018*
	No	4 (25.0)	276 (57.1)	
Received blood transfusion	Yes	0 (0)	23 (4.7)	1.000
	No	16 (100)	461 (95.3)	
Donated blood previously	Yes	15 (93.7)	437 (90.3)	1.000
	No	1 (6.3)	47 (9.7)	
Skin tattoo	Yes	1 (6.3)	52 (10.7)	1.000
	No	15 (93.7)	432 (89.3)	

^a^ All data presented as number of blood donors (%). Odds ratios for significant descriptors are provided in Table 2.

^b^ Univariate analysis performed with Fisher’s exact test or Fisher-Freeman-Halton test if more than 2 rows and 2 columns were analyzed.

^c^ The Brazilian monthly minimal wage was US$ 244 at the time of writing. Detailed data set can be reviewed under S1 Dataset.

*Results statistically significant.

**Table 2:** Factors associated with occurrence of *Bartonella* infection in blood donors in Campinas, Brazil.

Risk factor	Univariate^a^			Multivariate^b^		
	OR	95% CI	p-value	Adjusted OR	95% CI	*p* value
Occupational animal exposure	7.2	1.4–36.3	0.048	N/A^c^	N/A	N/A
Contact with cats	3.8	1.3–10.8	0.018	3.4	1.1–9.6	0.033
Past tick bite	4.0	1.3–12.6	0.018	3.7	1.3–13.4	0.017

OR, odds ratio

^a^ Univariate analysis performed with Fisher’s exact test.

^b^Multivariate analysis performed by logistic regression.

^c^ This variable was not maintained in the final multivariate model due to collinearity with the variable “contact with cats”. Detailed data set can be reviewed under S1 Dataset.

## Discussion

This study identified two risk factors associated with subclinical *Bartonella* species bloodstream infections in blood donors. *Bartonella* species infection was three times more likely to be diagnosed in blood donors who had contact with cats compared to blood donors with no contact. Similarly, blood donors working in animal-related professions were seven times more likely to be infected with these pathogenic bacteria, although this variable was not included in the multivariate model due to collinearity. In Swedish blood donors, contact with cats was also a risk factor for *B*. *elizabethae* seropositivity [7]. Our findings indicate that cat contact also increases the risk of subclinical bloodstream infections with *B*. *henselae* or *B*. *clarridgeiae*. Previously, 24% of 192 non-immunocompromised Americans with frequent exposure to cats, cat scratches or fleas had detectable *Bartonella* species DNA in blood specimens using the same diagnostic approach as used in our study [9]. Cats are natural reservoir hosts for *B*. *henselae*, *B*. *clarridgeiae*, and *Bartonella koehlerae*, all of which are important zoonotic species [2]. After flea-transmitted infection with most *Bartonella* species strains, cats rarely develop clinical manifestations but remain persistently infected with high levels of intravascular bacteria, which facilitates pathogen acquisition by blood-sucking vectors [10]. Recently, artificial feeding of *Ctenocephalides felis* with *B*. *henselae-* or *B*. *clarridgeiae-* infected blood demonstrated that these pathogens can persist in *C*. *felis* and be excreted in flea feces [14]. Cat nails can be contaminated with infected flea feces, where *B*. *henselae* can survive for several days [15]. Needle stick transmission of *Bartonella* species has also been reported [16]. Bacterial transmission is less likely to occur by cat bite, since shedding of *Bartonella* species in cat saliva has not been clearly documented [17]. Interestingly, a history of cat bite, cat scratches, or flea bite was not significantly associated with *Bartonella* species infection in our study. Possible explanations include donors avoiding cat bites and scratches during “rough play”, lack of identifying fleas as the source of an insect bite, cat confinement to the house, and routine use of parasiticides.

In Southeast Brazil, the combined *Bartonella* prevalence reported in domestic and stray cats by five reports was 38.5% (112/291), but ranged from 4.3% to 97% among feline populations tested [18, 19, 20, 21, 22]. Such wide variation may be associated with level of flea infestation or analytical sensitivity of PCR assays used. *B*. *henselae* and *B*. *clarridgeiae* were the only two species detected by molecular methods from cats in these studies. Furthermore, we have tested 112 cats from the same county of the blood donors (Campinas, Brazil) for *Bartonella* bacteremia, using the same diagnostic approach used in the present report (BAPGM culture and PCR), where we detected bacteremia in 90% (101/112) of cats and *B*. *henselae* was confirmed by DNA sequencing and BLAST analysis from three culture isolates [23]. Therefore, similar to reports from other countries, cats may be the main reservoirs of *B*. *henselae* and *B*. *clarridgeiae* in Southeast Brazil, and pose as a risk factor for subclinical infection in humans in this region.

A previous history of tick bite was also determined to be a risk factor for *Bartonella* species infection in this human population. While cat fleas are a well-established vector, the capability of ticks to transmit these organisms has been the subject of substantial debate. It has been demonstrated that seven *Bartonella* species are capable of growing in an *Amblyomma americanum* tick cell line [24]. Recently, the vector competency of *Ixodes ricinus* for transmission of *Bartonella birtlesii* was confirmed using a mouse model [25]. Although. *Ixodes ricinus* and *Amblyomma americanum* are not present in Brazil, other ticks such as *Ixodes loricatus*, *Ixodes didelphidis*, *Amblyomma cajennense*, *Amblyomma aureolatum*, *Amblyomma ovale*, and *Rhipicephalus sanguineus* have been isolated from marsupials and rodents living in a locality where other tick-borne organisms had been previously identified [26]. Therefore, while the mode of transmission of any *Bartonella* species to humans is still unclear, the potential role of tick transmission should not be ignored.

Cat-scratch disease, bacillary angiomatosis and endocarditis are the most frequently reported manifestation of *Bartonella* infection in both immunocompetent or immunocompromised Brazilian populations [27–29], although ocular, neurologic and dermatologic abnormalities were also reported [30]. In addition, Correa *et al*. reported that the risk of *Bartonella* DNA bloodstream infection was 45 times higher in arrhythmic patients from Brazil or Argentina when compared to controls [31]. Our results expand the current understanding about *Bartonella* species infection in humans in Brazil by reinforcing the ecological role of cats and ectoparasites in the transmission of *Bartonella*. Risk of human infection can be minimized by implementing year-round ectoparasite control in domestic animals, by avoiding cat bites and scratches, and by keeping cats indoors to minimize exposure to vectors [32]. The medical impact of *Bartonella* species occurrence in the blood supply is still unknown and should be carefully investigated.

## Supporting Information

S1 Dataset Complete data set of Brazilian blood donors who participated in the study. Donors are identified by a unique numerical ID, and significantly associated variables (Tables 1 and 2) are provided with responses for occupational animal exposure, contact with cats, and recall of a past tick bite. Column labeled “status” refers to presence or absence of *Bartonella* infection following blood sample analysis described in Materials and Methods.
(XLSX)
